# Supplementary material for: Elucidating the mechanism of heterogeneous Wacker oxidation over Pd-Cu/zeolite Y by transient XAS
Source: Nat Commun. 2020 Feb 28;11:1118. doi: 10.1038/s41467-020-14982-x (PMC7048791; doi:10.1038/s41467-020-14982-x)
Supplement: Supplementary file 1 — Supplementary Information [file 41467_2020_14982_MOESM1_ESM.pdf]

Supplementary Information

Elucidating the mechanism of heterogeneous Wacker oxidation over Pd-Cu/zeolite Y by transient XAS

Imbao et al.

Contents

Supplementary Methods 3
Supplementary Figures 6
Supplementary Tables 20
Supplementary Discussion 22
  Catalyst characterization 22
  Wacker oxidation of ethylene 23
  PCA and MCR-ALS Results 24
    a. TPR-XANES of Cu7Y and Pd2-Y 24
    b. Principal component analysis 25
    c. MCR-ALS analysis 25
Supplementary References 27

Supplementary Methods

Elemental Analysis. The amount of copper and palladium were determined by means of atomic absorption spectroscopy (AAS, SpectrAA 220FS). Twenty milligrams of the metal-exchanged zeolite catalysts were digested in a solution containing 2 mL hydrofluoric acid (HF) and 3 mL nitric acid (HNO_3) and diluted 50 times before measurement. The carbon content of the as-prepared and spent catalysts was quantified through the combustion products of carbon (CO_2) via infrared spectroscopy using LECO TruSpec Micro.

Surface Area and Pore Volume Determination. All the zeolite samples were evacuated at 523 K to less than 0.02 mbar in vacuum before performing N_2 physisorption experiments in a Micromeritics Tristar II 3020 analyzer at liquid nitrogen temperature (77 K).

X-ray Diffraction. Powder XRD patterns were acquired by utilizing a Bruker D8 diffractometer using Cu-K_α radiation (λ = 1.5418 Å, 40 kV, 40 mA). The XRD patterns were collected in the range of 4° to 60° in 2θ with a step size of 0.02°.

Temperature-Programmed Reduction. TPR experiments were done on a Micromeritics AutoChem II 2920 instrument equipped with a thermal conductivity detector (TCD). The as-prepared samples were pre-treated in O_2 at 378 K for an hour and cooled to room temperature. Subsequently, the samples were heated from 298 to 823 K (5 K/min) and held at 823 K for 15 min in a 10% H_2/Ar mixture, and hydrogen consumption was recorded.

Electron Microscopy. Scanning transmission electron microscopy (STEM) was employed to determine the particle distribution in the metal-exchanged zeolite samples. STEM images of the samples were taken with a high-angle annular dark field (HAADF) detector performed on an aberration-corrected Hitachi HD2700CS

microscope at 200 kV. This was combined with energy-dispersive X-ray (EDX) spectroscopy allowing the determination of the elemental composition of a selected spot in the sample.

Attenuated total reflection infrared (ATR-IR) spectroscopy. ATR-IR spectroscopy was utilized to characterize the metal-exchanged zeolites by identification of the functional groups present. Spectra were recorded using the Bruker PLATINUM Diamond ATR unit and VERTEX 70v spectrometer equipped with a mercury cadmium telluride (MCT) detector cooled with liquid nitrogen. Spectra (4000–400 cm^{-1}) were obtained by averaging 100 scans at 4 cm^{-1} resolution.

XAS Data Processing/Treatment. The raw binary format QuickXAS data was processed using a python-based program developed in-house. Energy calibration was performed by consideration of the maximum derivative of Cu and Pd foils, which were measured simultaneously. Edge step normalization was performed using a 1st order polynomial pre-edge function and a Victoreen post-edge function (Supplementary Equation 1):

$$y = \frac{af^3}{E^3} - \frac{bf^4}{E^4} + c \tag{1}$$

where *E* is the energy; *a*, *b* and *c* are Victoreen coefficients; and $f = 1.23986 \times 10^4$ / Å V.

To reduce data-point density from extreme oversampling, a localised radial basis function interpolation was performed with a constant energy step in the XANES region of 0.25 eV for Cu and 0.5 eV for Pd K-edge data and a constant k-step of 0.025 Å^{-1} in the EXAFS region for both edges.

EXAFS data were fitted using the Demeter software[1]. The Fourier transformation of the k^3-weighted Cu and Pd K-edge EXAFS data was done between 3-11 Å^{-1} and fitted in R space in the range of 1-2 Å for Cu and 1-3 Å for Pd. The theoretical phase shifts

and scattering amplitudes of the Cu-O, Pd-O and Pd-Pd shells were calculated by the FEFF6 code[2]. These references were used for the initial guess of the EXAFS fit for the following shells:

Cu-O: ICSD-16025 (CuO)

Pd-O: ICSD-257583 (PdO)

Pd-Pd: ICSD-77885 (Pd)

Supplementary Figures

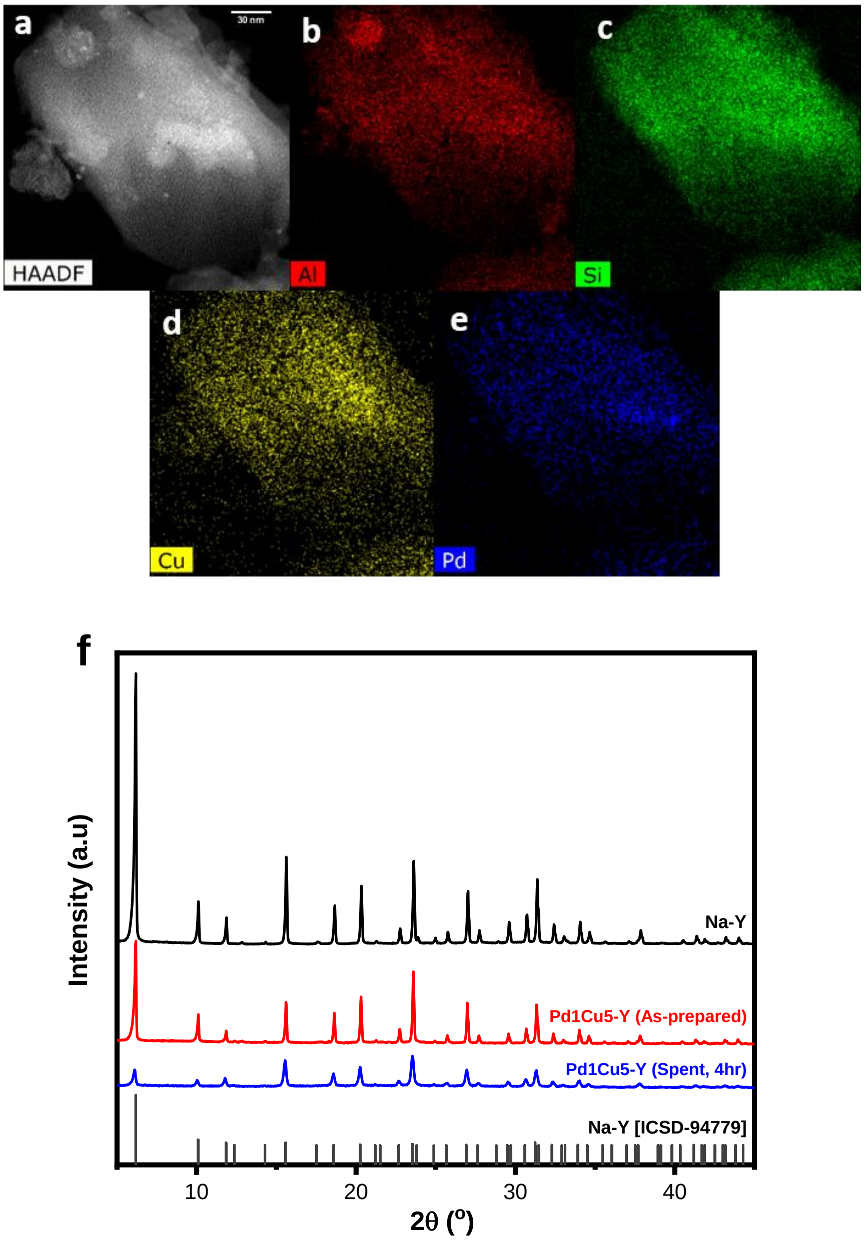

Supplementary Figure 1 | Catalyst characterization. (a) HAADF-STEM image and the corresponding **(b-e)** EDX elemental maps of as-prepared Pd1Cu5-Y. Scale bar: 30 nm. **(f)** XRD patterns of parent Na-Y zeolite, as-prepared and spent Pd1Cu5-Y.

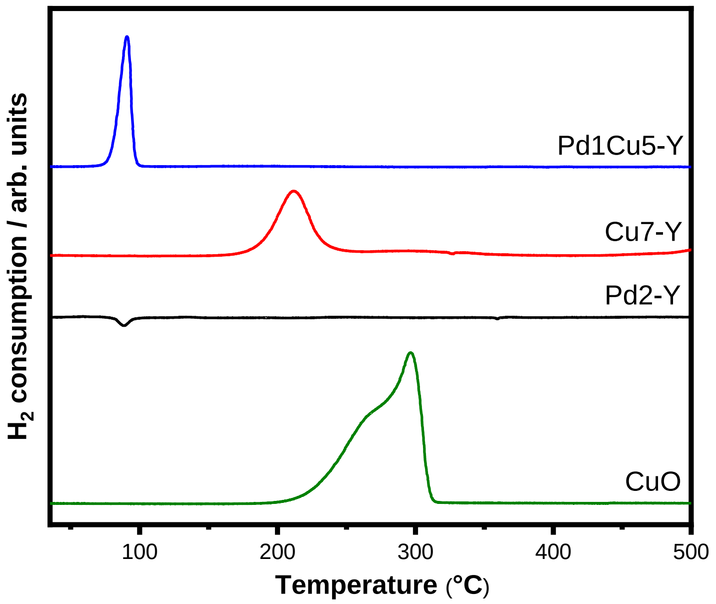

Supplementary Figure 2 | Temperature-programmed reduction. TPR profiles of the metal-exchanged zeolite Y supported catalysts and the CuO reference.

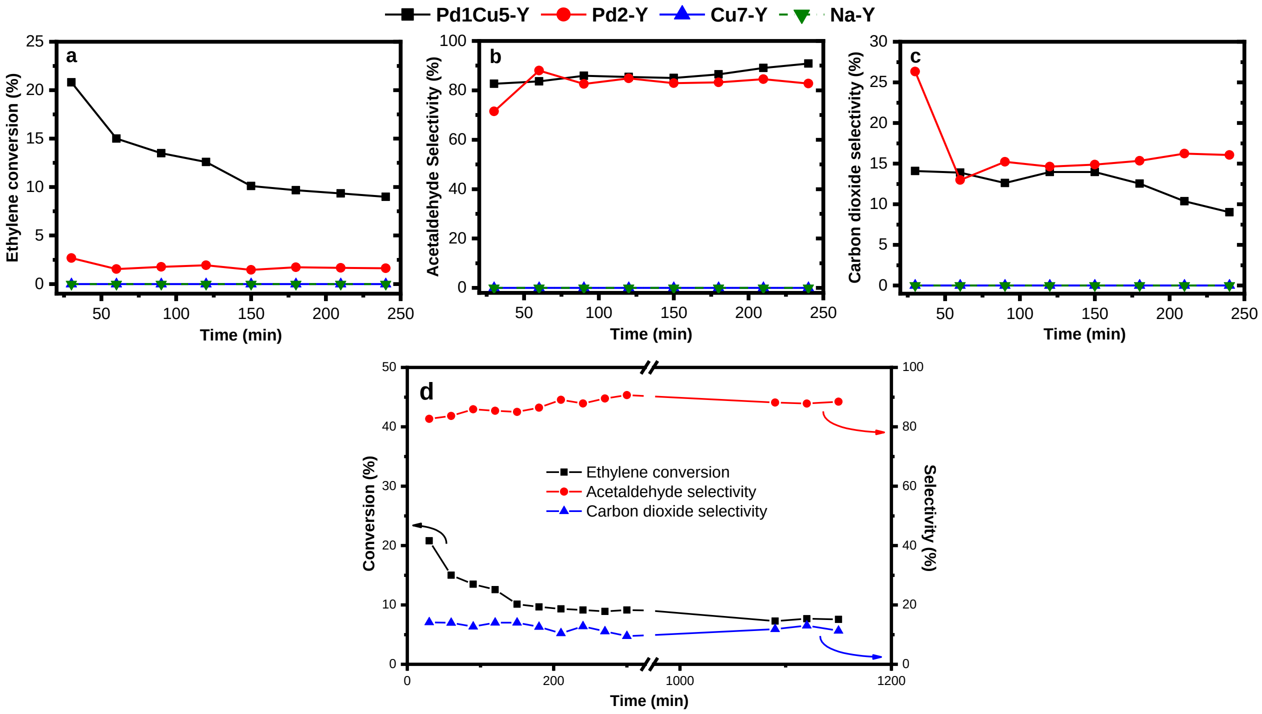

Supplementary Figure 3 | Ethylene oxidation over ion-exchanged zeolite Y. (a) Ethylene conversion and **(b,c)** product selectivities over Pd1Cu5-Y, Pd2-Y, Cu7-Y and Na-Y vs time on stream (TOS). Na-Y and Cu7-Y are inactive towards Wacker oxidation under the standard reaction conditions used (378 K, 1 atm, $W/F_0 = 0.86$ kg_{cat} s mol^{-1}). **(d)** Stability test: ethylene conversion and product selectivities *vs* time on stream over Pd1Cu5-Y.

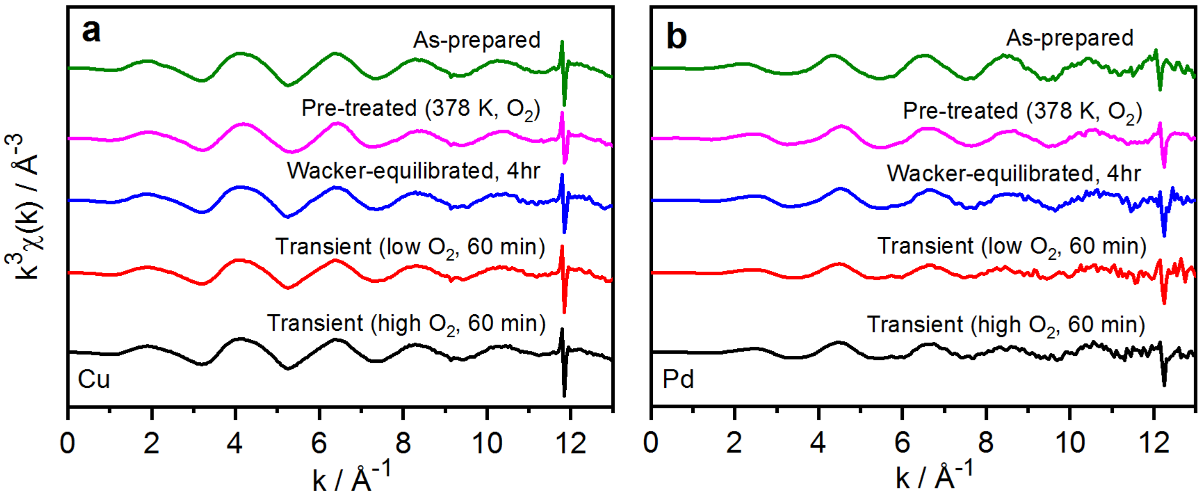

Supplementary Figure 4 | *In situ* EXAFS spectra. k^3-weighted (**a**) Cu and (**b**) Pd K-edge EXAFS spectra of Pd1Cu5-Y before and during transient experiments.

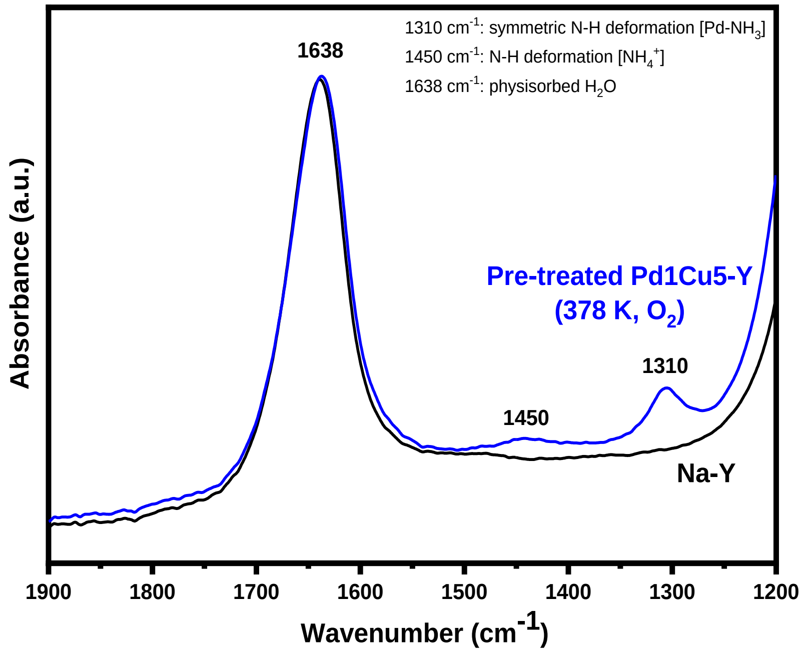

Supplementary Figure 5 | ATR-IR spectroscopy. ATR-IR spectra of parent zeolite Na-Y and pre-treated Pd1Cu5-Y.

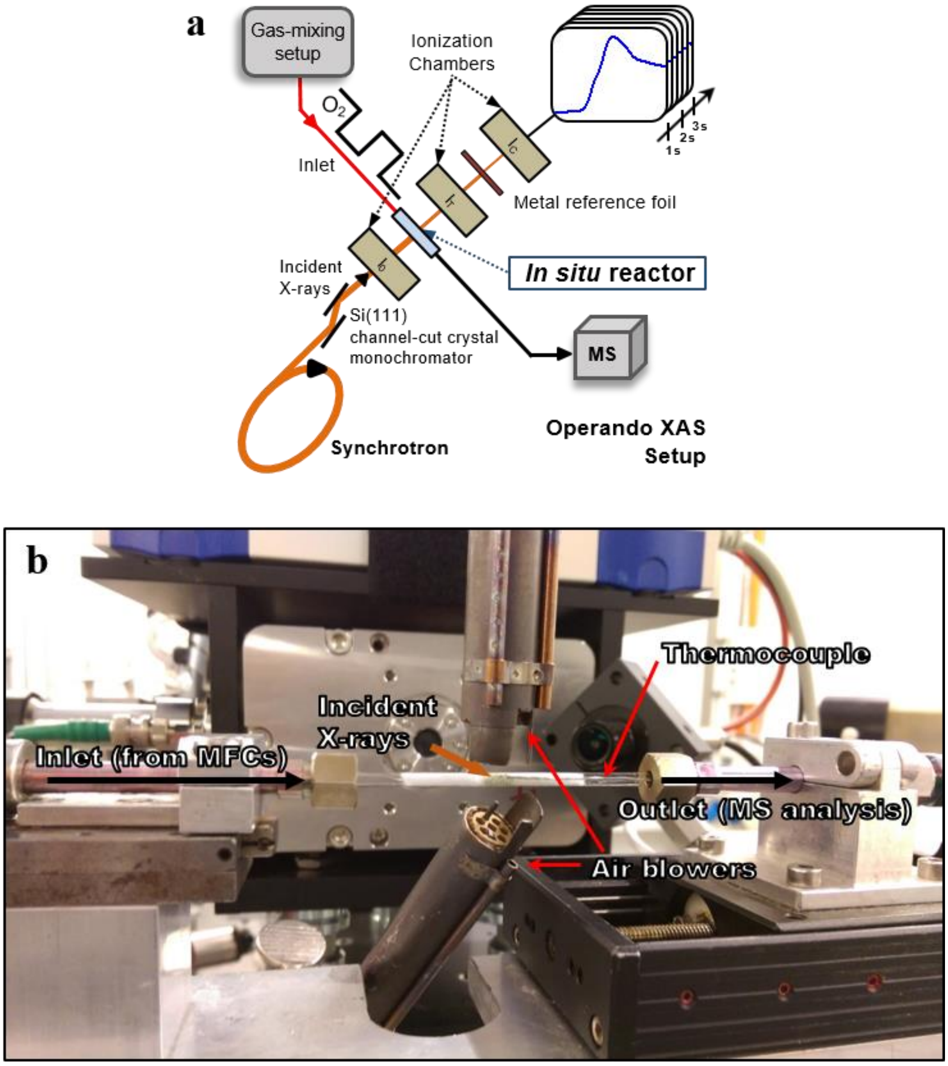

Supplementary Figure 6 | Operando XAS setup. (a) Experimental setup scheme for operando XAS measurements and **(b)** the *in situ* setup with the actual capillary reactor used.

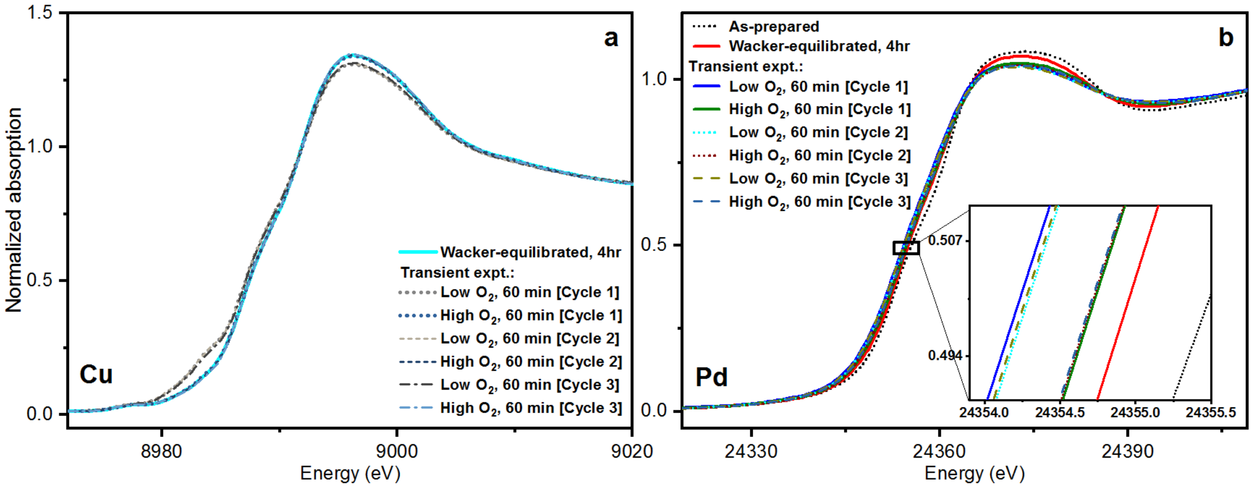

Supplementary Figure 7 | *In situ* XANES spectra of Pd1Cu5-Y. Normalized **(a)** Cu and **(b)** Pd K-edge XANES spectra of Pd1Cu5-Y at the end of each kinetic regime switching during the transient experiments under varying O_2 partial pressure.

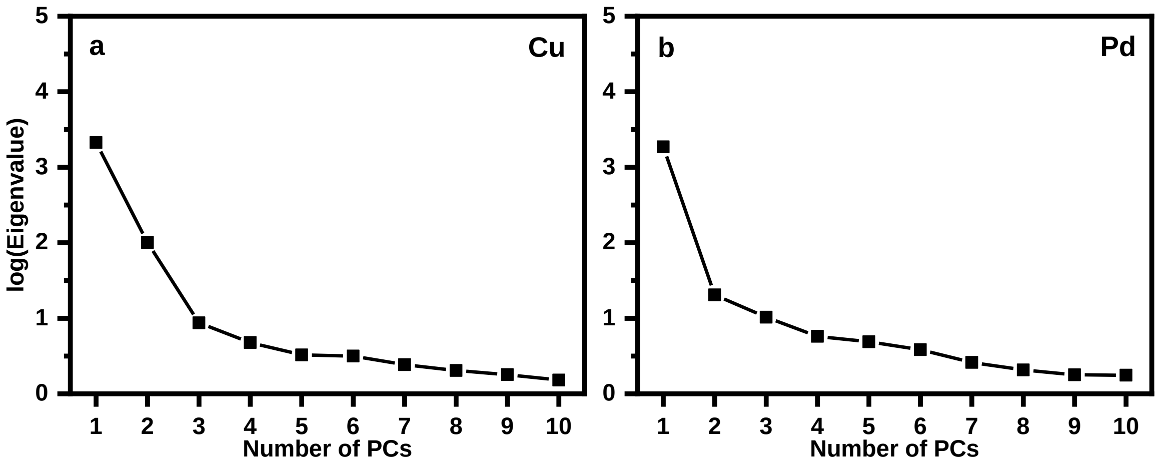

Supplementary Figure 8 | Principal Component Analysis. Scree plots from PCA of the **(a)** Cu and **(b)** Pd K-edge XANES of Pd1Cu5-Y during all experiments.

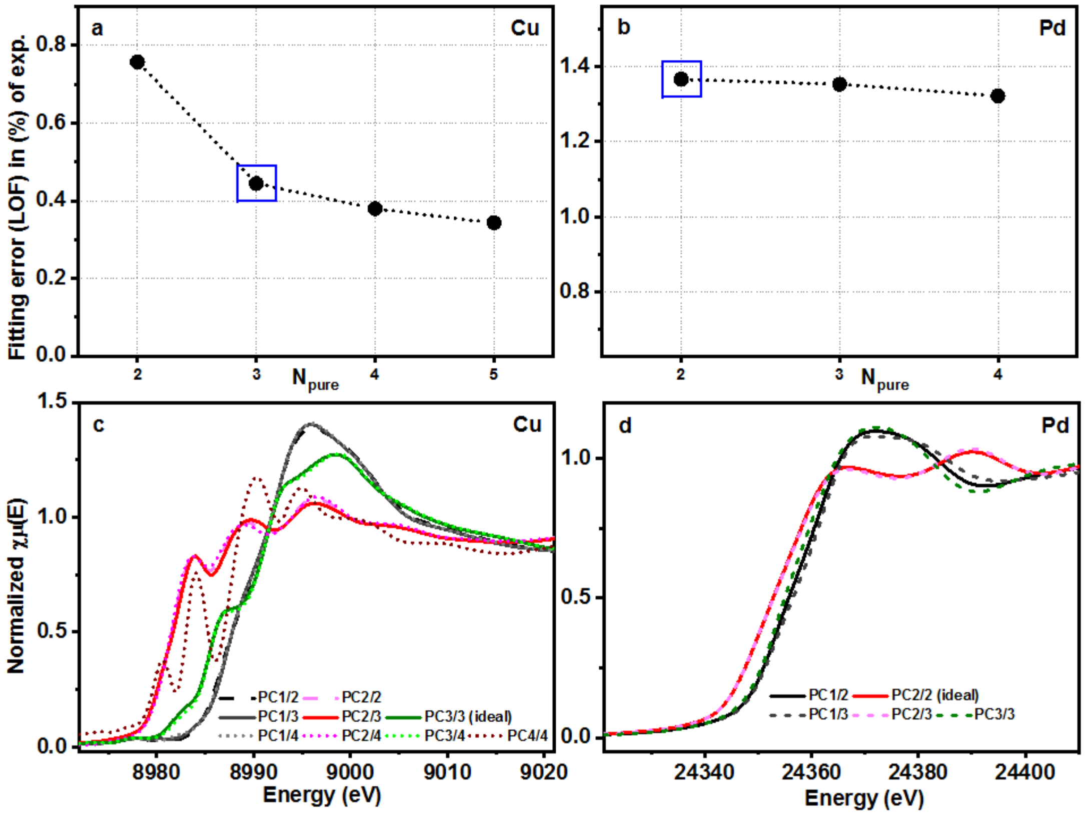

Supplementary Figure 9 | MCR-ALS Analysis. (a,b) Fitting error as a function of N_{pure} and **(c,d)** the XANES spectra of pure components derived from MCR-ALS using different N_{pure} for Cu and Pd. The boxed points in **(a,b)** indicate the optimal N_{pure} for Cu and Pd, respectively.

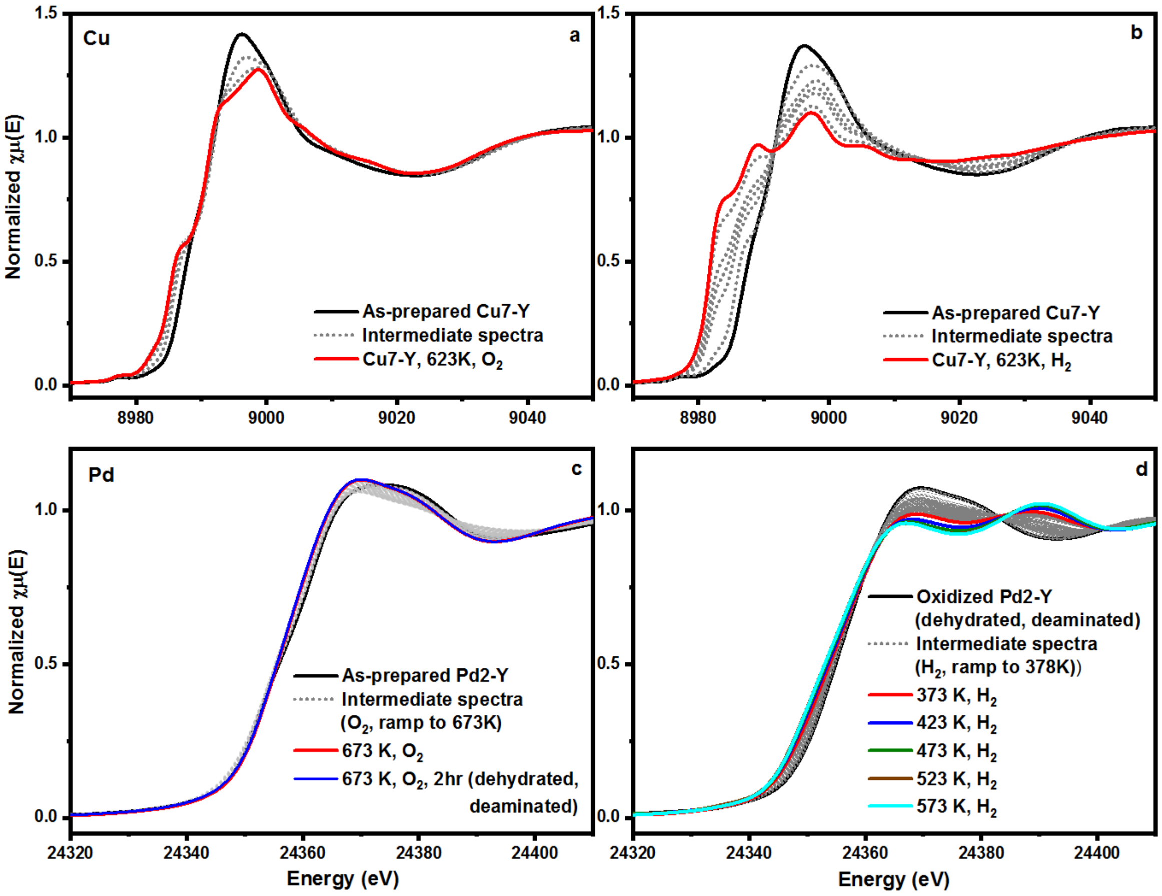

Supplementary Figure 10 | TPR-XANES of Cu7Y and Pd2-Y. *In situ* **(a,b)** Cu and **(c,d)** Pd K-edge XANES of Cu7-Y and Pd2-Y during temperature-programmed reaction under **(a,c)** oxygen and **(b,d)** hydrogen. All spectra are normalized.

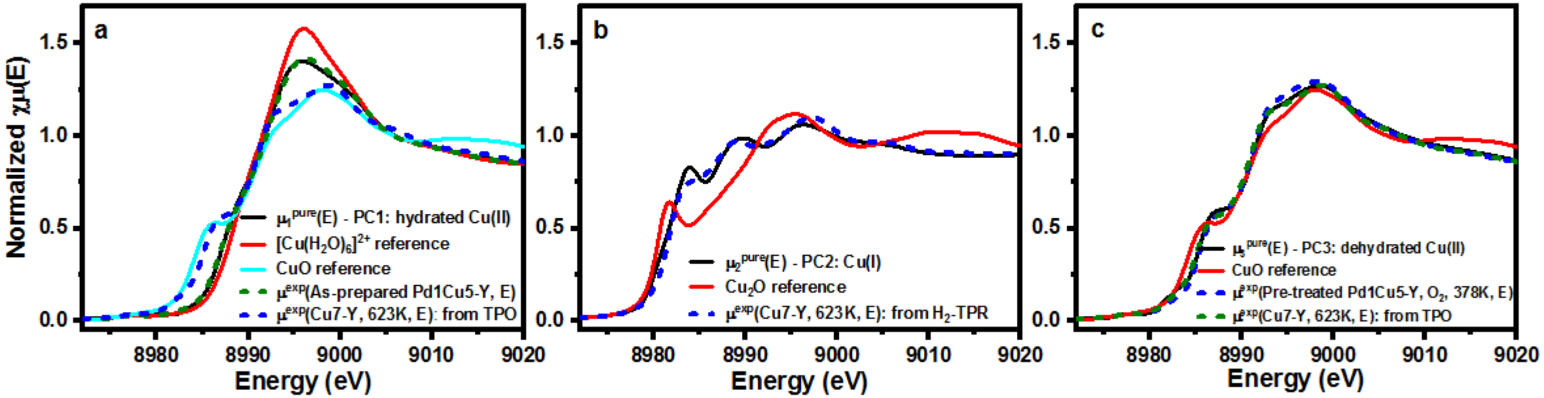

Supplementary Figure 11 | Assignment of MCR-ALS $\mu^{pure}(E)$ spectra for Cu. Comparison of the MCR-ALS-derived pure spectra with experimental references: **(a)** PC1, **(b)** PC2, and **(c)** PC3.

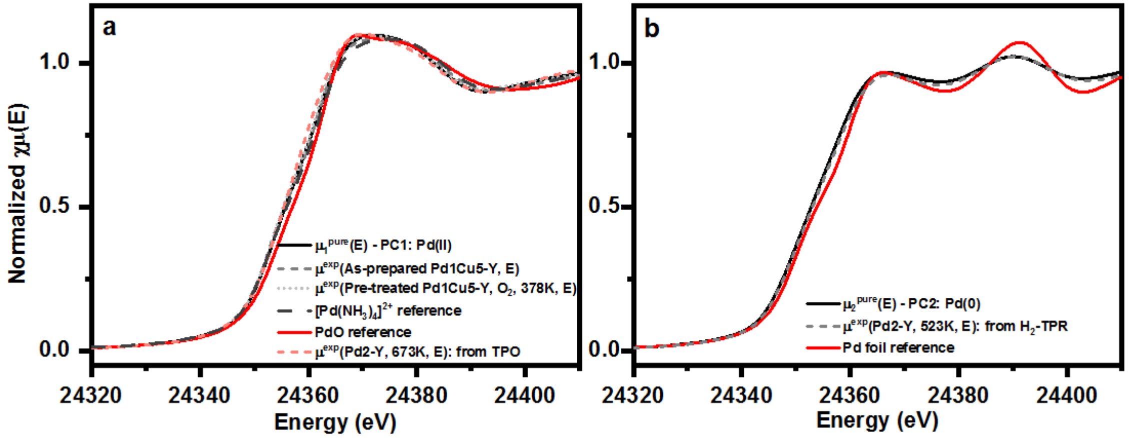

Supplementary Figure 12 | Assignment of MCR-ALS $\mu^{pure}(E)$ spectra for Pd. Comparison of the MCR-ALS-derived pure spectra with experimental references: **(a)** PC1 and **(b)** PC2.

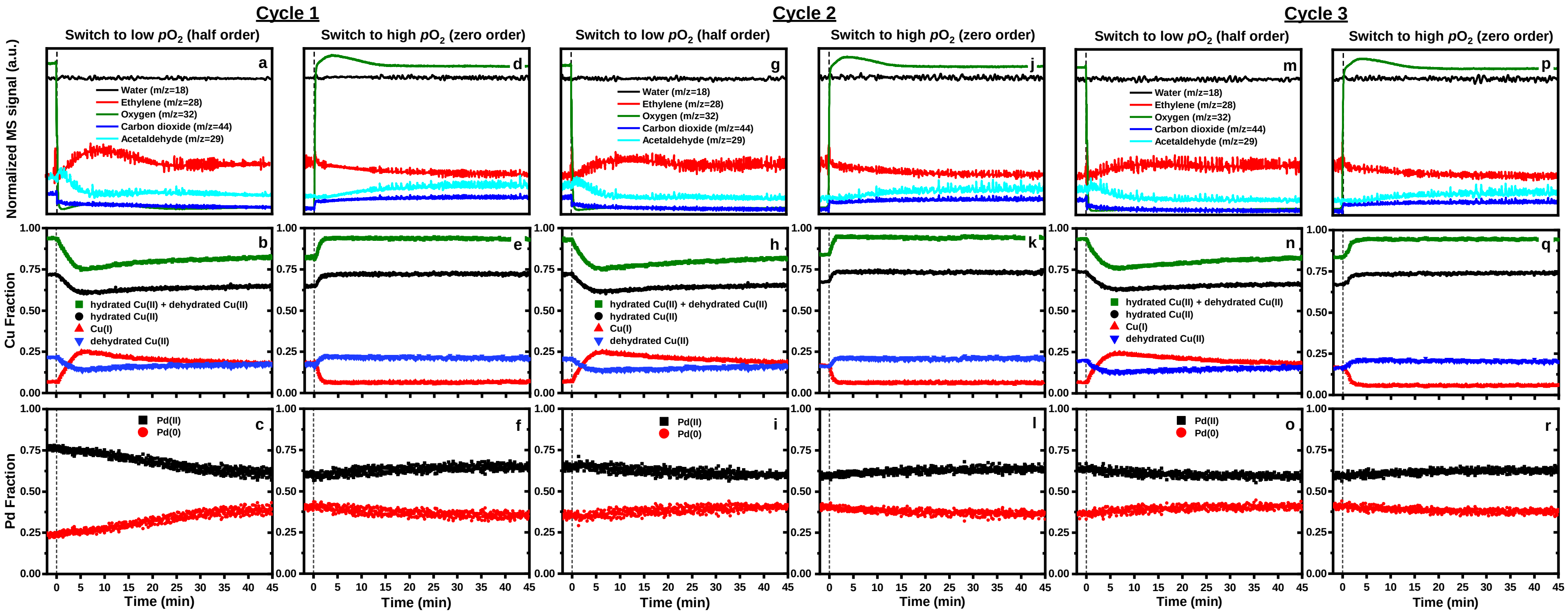

Supplementary Figure 13 | Transient oxygen switching experiments. Normalized mass spectrometry (MS) signals (**a,d**) of outlet gas components as a function of time after changing the partial pressure of O_2 in the feed. Corresponding dynamic copper (**b,e**) and palladium (**c,f**) speciation calculated from MCR-ALS of time-resolved XANES. The catalyst was equilibrated under Wacker conditions for 4 hours before the first change in O_2 partial pressure (t<0). After changing the partial pressure of O_2 (zero order to half order and back), the catalyst was subjected to the gas feed (shown here is the first 45 min) until the MS signals equilibrated. This transient experiment was done two more times: Cycle 2 (**g-l**, Supplementary Fig. 15) and Cycle 3 (**m-r**, Supplementary Fig. 16).

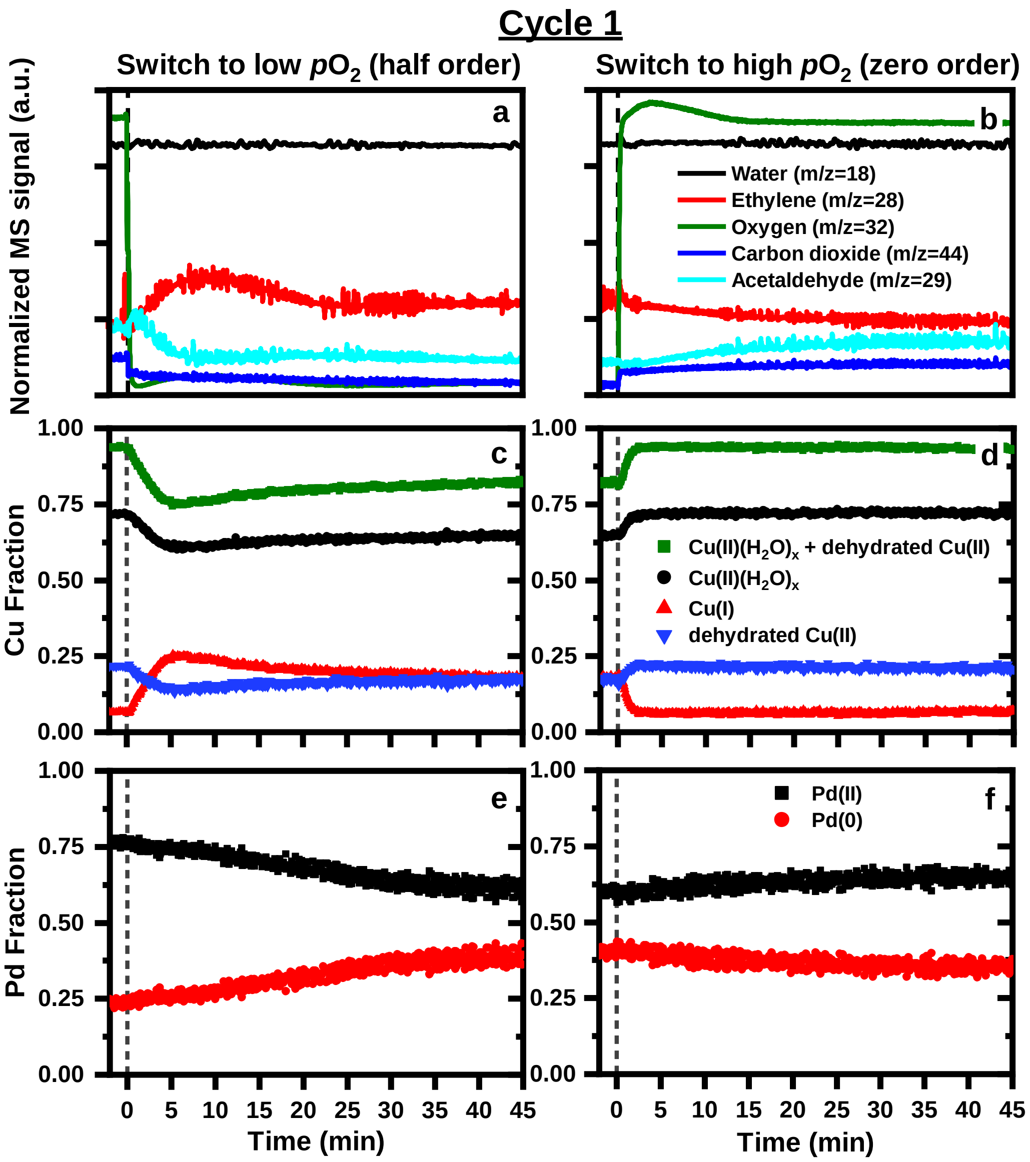

Supplementary Figure 14 | Cycle 1: Transient oxygen switching experiment. Normalized mass spectrometry (MS) signals **(a,d)** of outlet gas components as a function of time after changing the partial pressure of O_2 in the feed. Corresponding dynamic copper **(b,e)** and palladium **(c,f)** speciation calculated from MCR-ALS of time-resolved XANES. The catalyst was equilibrated under Wacker conditions for 4 hours before the first change in O_2 partial pressure (t<0). After changing the partial pressure of O_2 (zero order to half order and back), the catalyst was subjected to the gas feed (shown here is the first 45 min) until the MS signals equilibrated.

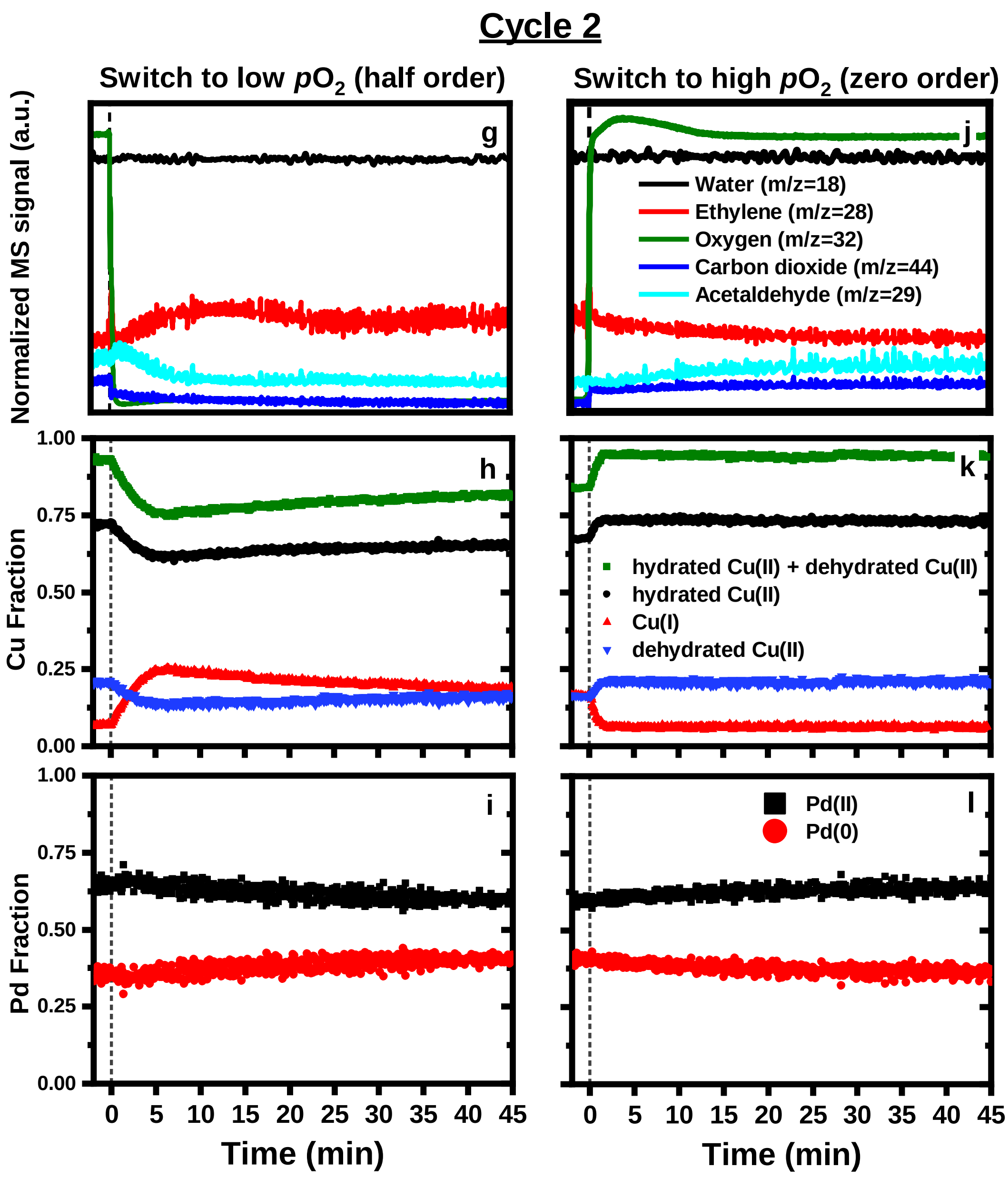

Supplementary Figure 15 | Cycle 2: Transient oxygen switching experiment. Normalized mass spectrometry (MS) signals **(g,j)** of outlet gas components as a function of time after changing the partial pressure of O_2 in the feed. Corresponding dynamic copper **(h,k)** and palladium **(i,l)** speciation calculated from MCR-ALS of time-resolved XANES. After changing the partial pressure of O_2 (zero order to half order and back), the catalyst was subjected to the gas feed (shown here is the first 45 min) until the MS signals equilibrated.

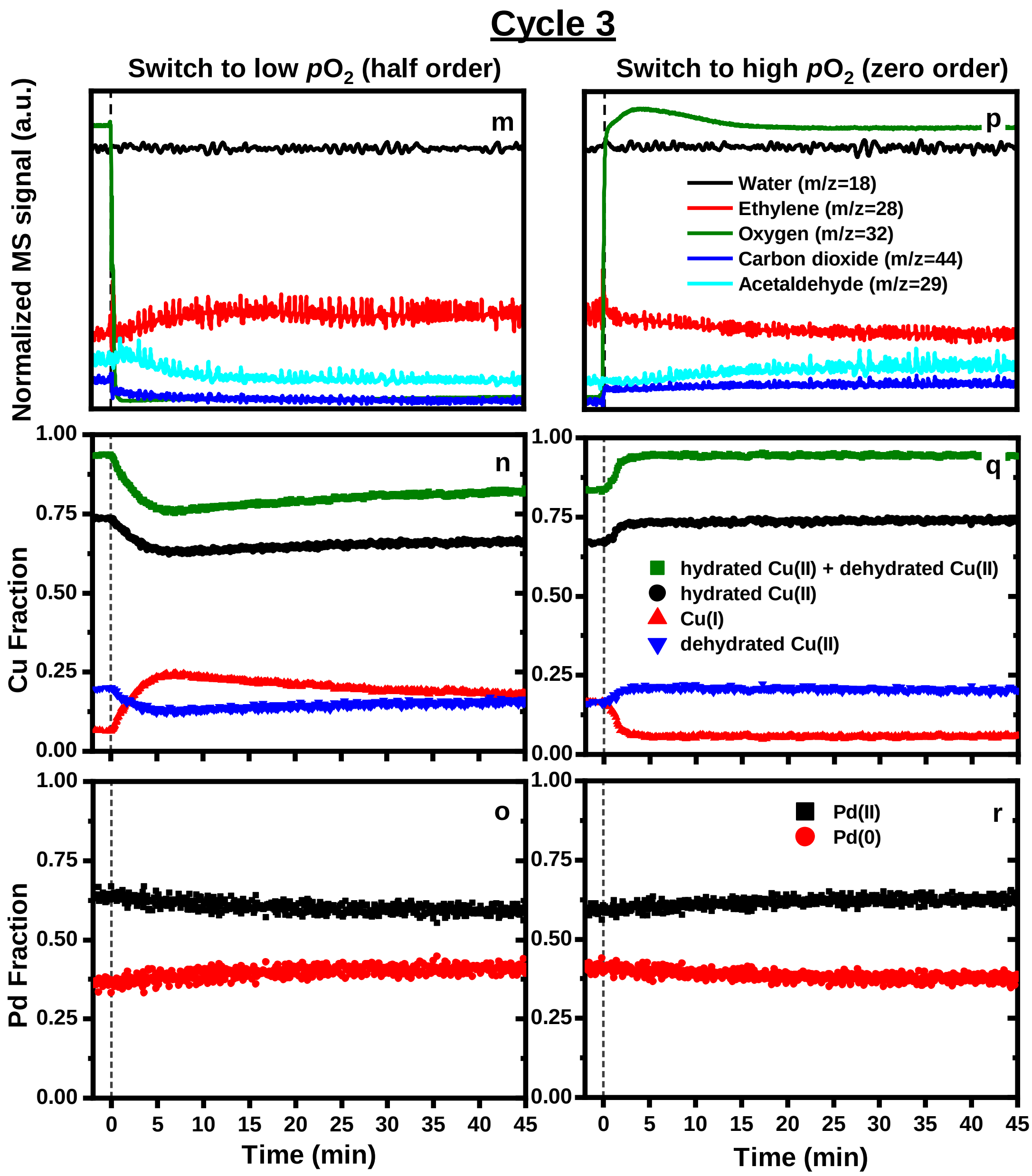

Supplementary Figure 16 | Cycle 3: Transient oxygen switching experiment. Normalized mass spectrometry (MS) signals **(m,p)** of outlet gas components as a function of time after changing the partial pressure of O_2 in the feed. Corresponding dynamic copper **(n,q)** and palladium **(o,r)** speciation calculated from MCR-ALS of time-resolved XANES. After changing the partial pressure of O_2 (zero order to half order and back), the catalyst was subjected to the gas feed (shown here is the first 45 min) until the MS signals equilibrated.

Supplementary Tables

Supplementary Table 1 | Cu and Pd contents (determined by AAS of totally dissolved as-prepared catalysts) of catalysts used.

Sample	Cu (wt%)	Pd (wt%)	Cu/Pd
Cu7-Y	6.7	Not measured	
Pd2-Y	Not measured	1.9	
Pd1Cu5-Y	4.7	1.1	6.7

Supplementary Table 2 | Properties of the parent Na-Y zeolite and the ion-exchanged catalysts before and after reaction.

Sample	S_{BET} (m^2 g^{-1})[a]	V_{total} (cm^3 g^{-1})[b]	V_{micro} (cm^3 g^{-1})[c]	V_{meso} (cm^3 g^{-1})[d]	Carbon (wt%)[e]
Na-Y	660	0.34	0.32	0.019	Not measured
Pd1Cu5-Y (as-prepared)	612	0.31	0.31	0.007	0.1
Pd1Cu5-Y (spent)[f]	411	0.21	0.21	0.004	5.4

(a) S_{BET}: surface area determined by Brunauer-Emmett-Teller theory; **(b)** Total pore volume = adsorbed volume at P/P_0 = 0.95; **(c)** Micropore volume obtained by the t-plot method; **(d)** Mesopore volume = V_{total} – V_{micro}; **(e)** From infrared spectroscopy; **(f)** Measured after 4 hr under Wacker conditions.

Supplementary Table 3 | Fraction of Pd and Cu and the rate of acetaldehyde and carbon dioxide formation in the three oxygen switching cycles.

Fraction[a]	t=0	Cycle 1		Cycle 2		Cycle 3	
		Low pO_2	High pO_2	Low pO_2	High pO_2	Low pO_2	High pO_2
Pd(II)	0.76	0.60	0.64	0.60	0.63	0.60	0.64
Cu(II)	0.94	0.83	0.93	0.83	0.93	0.83	0.94

Relative rate of formation[b]	t=0	Cycle 1		Cycle 2		Cycle 3	
		Low pO_2	High pO_2	Low pO_2	High pO_2	Low pO_2	High pO_2
Acetaldehyde	1.0	0.26	0.57	0.24	0.59	0.24	0.57
Carbon dioxide	1.0	0.10	0.85	0.09	0.88	0.09	0.87

(a) The values of the average fitting error derived from MCR-ALS analysis are reported in Supplementary Fig. 9; **(b)** Relative to the rate of formation before the start of transient experiments (t=0, equilibration under standard Wacker conditions after 4 hr).

Supplementary Discussion

Catalyst characterization

Supplementary Fig. 1 shows the XRD patterns of the parent zeolite Na-Y and Pd1Cu5-Y, revealing only the typical reflections attributed to the crystalline zeolite, indicating that the main crystalline features of the zeolite phase are well-preserved after ion exchange. Reflections ascribed to crystalline metallic palladium and copper and their oxides are not present, suggesting the absence of such particles larger than 3 nm.

The BET surface area and micropore volume of the as-prepared catalyst (Supplementary Table 2) indicate high crystallinity of the zeolite phase, which decreased slightly after ion exchange. A further significant reduction in the surface area and pore volume after Wacker oxidation is also evident, which might have originated from the accumulation of carbon deposits, which increased from 0.1 wt.% in the as-prepared catalyst to 5.4 wt.% in the spent catalyst.

Supplementary Fig. 2 depicts the temperature-programmed reduction (TPR) profiles of Cu7-Y, Pd2-Y, Pd1Cu5-Y and cupric oxide. The TPR profile of Pd2-Y reveals that palladium was already reduced at room temperature and only shows a hydrogen desorption peak at around 363-368 K, which is indicative of Pd-β hydride decomposition[3]. The sharp peak in the TPR profile of Cu7-Y at around 483 K corresponds to the reduction of Cu(II) to Cu(I) in the supercages of the zeolite, while the weak and broad peak centered at 573 K is associated with the reduction of Cu(II) situated in the less accessible sodalite cages[4]. The single peak feature in the TPR profile of the optimized catalyst, Pd1Cu5-Y, suggests that copper and palladium are in close contact. The presence of palladium in Pd1Cu5-Y yields a lower reduction temperature of Cu(II) (368 K) compared with that of Cu7-Y (483 K). Since the reduction of palladium starts at around 273 K, Pd(0) can instigate hydrogen spill-over to the

neighboring copper ions[5,6]. Hence, the presence of palladium in close proximity with copper resulted in copper reduction at lower temperature.

We also characterized the pre-treated catalyst with ATR-IR spectroscopy to confirm the presence of amminated Pd(II) (Supplementary Fig. 5). The weak band at 1310 cm^{-1} attributed to symmetric N-H deformation signifies the presence of amine coordinated to Pd(II). On the other hand, the absence of 1275 cm^{-1} band corresponding to the N-H deformation of $Cu(NH_3)_4^{2+}$ indicates that no ammonia was transferred from Pd(II) to Cu(II) after the ion exchange. The presence of a weak band at 1450 cm^{-1} indicates the release of some ammonia from Pd coordination[7].

Wacker oxidation of ethylene

Supplementary Fig. 3 shows the rate and selectivity of ethylene oxidation over ion-exchanged zeolite Y at 378 K. The ethylene conversion over Pd1Cu5-Y after 4 hours on stream is *ca.* 9% and the selectivity for acetaldehyde remained at around 90% for almost 20 hours on stream.

Over the copper-free Pd2-Y, conversion of ethylene is five times less than that over Pd1Cu5-Y and gradually decreases throughout the reaction with a selectivity of roughly 80% for acetaldehyde and 20% for carbon dioxide. The amount of acetaldehyde that is formed after 4 hours on stream, however, is more than the stoichiometric amount of palladium exchanged into the zeolite, inferring catalytic acetaldehyde formation. Without copper, the reduced palladium can be reoxidized by oxygen, albeit slowly, as observed in previous studies[8,9]. In the absence of palladium, no Wacker activity is observed over Na-Y and Cu7-Y.

Thus, the simultaneous presence of palladium and copper in the zeolite; and oxygen and water in the reactant feed is necessary to carry out the heterogeneous Wacker

oxidation of ethylene. Moreover, these results strongly suggest that palladium is the site of the partial oxidation of ethylene to acetaldehyde since both Cu7-Y and the Na-Y are inactive, corroborating previous studies reported on palladium-based Wacker catalysts[8-10].

PCA and MCR-ALS Results

Quantitative speciation of the time-resolved XANES dataset can be done by means of linear combination fit (LCF) analysis to separate different contributions in a multi-component series of spectra. However, accurate quantification can only be achieved with appropriate reference spectra. When investigating complex catalytic reactions, such as Wacker oxidation carried out with a palladium- and copper-based supported catalyst, using references of bulk compounds like metal foil and bulk oxides results in an unsatisfactory fit. Hence, XANES-TPR (temperature-programmed reaction) of Cu7-Y and Pd2-Y in oxidizing and reducing atmospheres were performed to serve as references for comparison instead of their bulk counterparts. Instead of LCF analysis, PCA was performed to determine number of principal components (PC) while MCR-ALS analysis was done to quantify the changes in the fractions of palladium and copper during the transient experiments.

a. TPO, TPR-XANES (temperature-programmed oxidation and reduction) of Cu7Y and Pd2-Y

Aside from the bulk references, the XANES spectra of oxidized and reduced Pd2-Y and Cu7-Y were obtained *in situ* (Supplementary Fig. 10). Exchanged $Pd(II)(NH_3)_4$ in zeolite Y is known to completely lose all its amine ligands and get fully oxidized at 673 K in oxygen atmosphere[11]. Flowing hydrogen at more than 373 K resulted in the total reduction of Pd(II) to Pd(0). Thus, oxidized Pd2-Y at 673 K and its subsequent

reduction at 573 K were used as references for Pd(II) and Pd(0) on zeolite Y, respectively. On the other hand, a fully-dehydrated oxidized Cu7-Y at 623 K and its reduced form at 623 K were used as references for comparison for Cu(II) and Cu(I) species.

b. Principal component analysis

Supplementary Fig. 8 depicts the Scree plots exhibiting the dependence of PC eigenvalues to the total number of PCs obtained after the PCA of all *in situ* Cu and Pd K-edge XANES spectra of Pd1Cu5-Y. Visual inspection of the Scree plots, albeit qualitative, reveals that there are at least 3 PCs for copper and 2 PCs for palladium because a further increase in the number of PCs does not generate a significant variation in the eigenvalues.

c. MCR-ALS analysis

Implementation of MCR-ALS algorithm. The initial guesses in MCR-ALS for the spectra of the pure components, $\mu_i^{pure}(E)$, were generated via SIMPLISMA (SIMPLe-to-use Interactive Self-modeling Mixture Analysis)[12], a *pure variable selection* method. Subsequently, an MCR-ALS iterative refinement of the $\mu_i^{pure}(E)$ was performed and the results are reported in Supplementary Fig. 9 showing the Cu and Pd XANES spectra of the pure components and their respective fit quality indicators.

In the MCR-ALS analysis, a non-negativity constraint was performed on the concentration profiles and matrix of reference spectra while a closure-to-1 constraint was used for the concentration profiles.

N_{pure} greatly affects the MCR-ALS results so the optimal number of pure components was selected based on the reconstruction results from downsizing and upsizing the number of PCs[13] (<N_{pure} ± 1>: 2-4 for copper; 2-3 for palladium). The reconstruction of the spectra of pure components at different N_{pure} values are shown in Supplementary

Fig. 9, together with their corresponding lack of fit (LOF)[14] associated with the elements of the residual matrix and original dataset (Supplementary Equation 2):

$$\%\mathrm{LOF} = \sqrt{\frac{\Sigma_{i,j} r_{i,j}^2}{\Sigma_{i,j} e_{i,j}^2}} \qquad (2)$$

r_{ij} and e_{ij} are the elements (i,j) of the residual matrix and experimental dataset, respectively.

As expected, LOF decreases as N_{pure} increases, signifying a better reconstruction quality. Consistent with the results from PCA, a saturation effect is evident when N_{pure} is further increased from 3 for copper and 2 for palladium.

As shown in Supplementary Fig. 9, the pure spectra at different N_{pure} values do not show significant changes, validating the stability of the reconstruction. For copper at N_{pure}=4, the spectrum of the 4th pure component (PC4/4) does not match any reference and does not resemble any chemically meaningful spectrum. In the case of palladium, reconstruction at N_{pure}=3 yielded the spectra of two components, μ_2^{pure}(E)-PC2/3 and μ_3^{pure}(E)-PC3/3, the average of which is the μ_2^{pure}(E)-PC2/2 retrieved from the optimal 2-PC model.

Assignment of MCR-ALS-derived $\mu^{pure}(E)$ spectra. Cu(II) species can be distinguished by looking at the pre-edge feature at ~8977.5 eV due to 1s→3d transition[14,15], which is present in PC1 and PC3. As shown in Supplementary Fig. 11, the spectrum of PC1 resembles the hexaaquacopper(II) ion reference with the characteristic peak at *ca.* 8996 eV associated with mobile and highly-coordinated Cu(II) aqua complexes. PC3 can be attributed to a dehydrated Cu(II), similar to the spectrum of Cu7-Y after being oxidized at 623 K in O_2 atmosphere. PC2 can easily be identified as the only Cu(I) species with a rising-edge peak at ~8983 eV[15,16].

The Pd K edge XANES spectra (Supplementary Fig. 12) derived from MCR-ALS show that PC1 can be assigned to the Pd(II) ions similar to the $[Pd(NH_3)_4]^{2+}$ precursor while the PC2 is attributed to Pd(0) resembling that of reduced Pd2-Y at 523 K from H_2-TPR. It is difficult to distinguish different Pd(II) species as there are no significant differences among them. Consequently, the two pure components from MCR-ALS analysis were just identified as Pd(II) and Pd(0).

Supplementary References

(1) Ravel, B. & Newville, M. ATHENA, ARTEMIS, HEPHAESTUS: data analysis for X-ray absorption spectroscopy using IFEFFIT. *J. Synchrotron Radiat.* **12**, 537–541 (2005).

(2) Zabinsky, S. I., Rehr, J. J., Ankudinov, A., Albers, R. C. & Eller, M. J. Multiple-scattering calculations of x-ray-absorption spectra. *Phys. Rev. B* **52**, 2995–3009 (1995).

(3) Batista, J., Pintar, A., Mandrino, D., Jenko, M. & Martin, V. XPS and TPR examinations of γ-alumina-supported Pd-Cu catalysts. *Appl. Catal., A.* **206**, 113–124 (2001).

(4) Gentry, S. J., Hurst, N. W. & Jones, A. Temperature programmed reduction of copper ions in zeolites. *J. Chem. Soc., Faraday Trans.* **75**, 1688 (1979).

(5) van der Heide, E., Zwinkels, M., Gerritsen, A. & Scholten, J. Oxidation of ethylene to acetaldehyde over a heterogenized surface-vanadate Wacker catalyst in the absence of gaseous oxygen. *Appl. Catal., A.* **86**, 181–198 (1992).

(6) Barthos, R., Novodárszki, G. & Valyon, J. Heterogeneous catalytic Wacker oxidation of ethylene over oxide-supported Pd/VO_x catalysts: the support effect. *React. Kinet. Mech. Cat.* **121**, 17–29 (2017).

(7) Espeel, P. H., De Peuter, G., Tielen, M. C. & Jacobs, P. A. Mechanism of the Wacker oxidation of alkenes over Cu-Pd-exchanged Y zeolites. *J. Phys. Chem.* **98**, 11588–11596 (1994).

(8) Arai, H., Yamashiro, T., Kubo, T. & Tominaga, H. The catalysis of palladium and cupric ion-exchanged zeolite for oxidation of ethylene. *J. Jpn. Pet. Inst.* **18**, 39-44 (1976).

(9) Kubota, T., Kumada, F., Tominaga H. & Kunugi, T. Oxidation of propylene over a Pd(II)-Cu(II)-Y zeolite catalyst. *Int. Chem. Eng.* **13**, 539-545 (1973).

(10) Minachev, Kh. M., Usachev, N. Ya., Rodin, A. P., Kalinin, V. P. & Isakov, Ya. I. Oxidation of ethylene and propylene into carbonyl compounds on zeolite catalysts. *Petrol. Chem. U.S.S.R.* **3**, 186-193 (1979).

(11) Homeyer, S. T. & Sachtler, W. M. H. Elementary steps in the formation of highly dispersed palladium in NaY I. Pd ion coordination and migration. *J. Catal.* **117**, 91–101 (1989).

(12) Windig, W. & Guilment, J. Interactive self-modeling mixture analysis. *Anal. Chem.* **63**, 1425–1432 (1991).

(13) Martini, A. et al. Composition-driven Cu-speciation and reducibility in Cu-CHA zeolite catalysts: a multivariate XAS/FTIR approach to complexity. *Chem. Sci.* **8**, 6836–6851 (2017).

(14) Jaumot, J., Gargallo, R., de Juan, A. & Tauler, R. A graphical user-friendly interface for MCR-ALS: a new tool for multivariate curve resolution in MATLAB. *Chemometrics Intell. Lab. Syst.* **76**, 101–110 (2005).

(15) Kau, L. S., Solomon, E. I. & Hodgson, K. O. XANES/EXAFS study of the copper active site in methanol synthesis catalyst. *J. Phys. Colloques* **47**, C8-289-C8-292 (1986).

(16) Kau, L. S., Spira-Solomon, D. J., Penner-Hahn, J. E., Hodgson, K. O. & Solomon, E. I. X-ray absorption edge determination of the oxidation state and coordination number of copper: Application to the type 3 site in *Rhus vernicifera* laccase and its reaction with oxygen. *J. Am. Chem. Soc.* **109**, 6433–6442 (1987).
